# Modelling steroidogenesis: a framework model to support hypothesis generation and testing across endocrine studies

**DOI:** 10.1186/s13104-018-3365-y

**Published:** 2018-04-24

**Authors:** Laura O’Hara, Peter J. O’Shaughnessy, Tom C. Freeman, Lee B. Smith

**Affiliations:** 1 MRC Centre for Reproductive Health, The Queen’s Medical Research Institute, 47 Little France Crescent, Edinburgh, EH16 4TJ UK; 2 The Roslin Institute and Royal (Dick) School of Veterinary Studies, The University of Edinburgh, Midlothian, EH25 9RG UK; 3 Institute of Biodiversity, Animal Health and Comparative Medicine, University of Glasgow, Glasgow, G61 1QH UK; 4 School of Environmental and Life Sciences, University of Newcastle, Callaghan, NSW 2308 Australia

**Keywords:** Steroidogenesis, Model, Diagram

## Abstract

**Objective:**

Steroid hormones are responsible for the control of a wide range of physiological processes such as development, growth, reproduction, metabolism, and aging. Because of the variety of enzymes, substrates and products that take part in steroidogenesis and the compartmentalisation of its constituent reactions, it is a complex process to visualise and document. One of the goals of systems biology is to quantitatively describe the behaviour of complex biological systems that involve the interaction of many components. This can be done by representing these interactions visually in a pathway model and then optionally constructing a mathematical model of the interactions.

**Results:**

We have used the modified Edinburgh Pathway Notation to construct a framework diagram describing human steroidogenic pathways, which will be of use to endocrinologists. To demonstrate further utility, we show how such models can be parameterised with empirical data within the software Graphia Professional, to recapitulate specific examples of steroid hormone production, and also to mimic gene knockout. These framework models support in silico hypothesis generation and testing with utility across endocrine endpoints, with significant potential to reduce costs, time and animal numbers, whilst informing the design of planned studies.

**Electronic supplementary material:**

The online version of this article (10.1186/s13104-018-3365-y) contains supplementary material, which is available to authorized users.

## Introduction

Steroid hormones are responsible for the control of a wide range of physiological processes such as development, growth, reproduction, metabolism, and aging. Mammalian steroidogenesis uses cholesterol as a starting substrate to produce the steroid hormone classes of androgens, estrogens, progestogens, corticosteroids and mineralocorticoids. The initial conversion of cholesterol to the major biologically active steroids takes place primarily in the gonads, adrenals and placenta, but further specific interconversions can take place in peripheral tissues due to local expression of other enzymes (reviewed in [1]).

Because of the variety and location of the components that take part in steroidogenesis it is a complex process to visualise and document. However, over the years many attempts have been made, and searching for ‘steroidogenesis’ in Google images provides examples of these results. Some diagrams are incomplete and focus only on the steroid products without representing the enzymes that produce them; others focus only on the products of one particular organ. Steroidogenic enzymes often have more than one name or symbol (for example the enzyme cytochrome p450 side-chain cleavage is often referred to as p450scc in older literature, but has the gene symbol *CYP11A1* in humans). Many steroidogenesis diagrams often use non-standard nomenclature, or interchange gene names across species when the gene is specific to one species. Almost all pathway diagrams of steroidogenesis have no way of incorporating references into the diagram reducing their value as a learning resource for the end user.

One of the goals of systems biology is to quantitatively describe the behaviour of complex biological systems that involve the interaction of many components. This can be done by representing these interactions visually in a pathway model and then optionally constructing a mathematical model of the interactions. Steroidogenesis would greatly benefit from a formalised graphical documentation using a standard notation with links to original research articles and chemical structures of steroid intermediates. If the diagram could be parameterised to form a dynamic mathematical model it could potentially be used to predict what steroid pathways would be active and what products could be made by tissues expressing a particular combination of steroidogenic enzymes.

In this paper, we have used the modified Edinburgh pathway notation (mEPN) to construct a framework diagram describing human steroidogenic pathways, which will be of use to endocrinologists. To demonstrate further utility, we show how such models can be parameterised with empirical data within the software Graphia Professional [2], to recapitulate specific examples of steroid hormone production, and also to mimic gene knockout. These framework models support in silico hypothesis generation and testing with utility across endocrine endpoints, with significant potential to reduce costs, time and animal numbers, whilst informing the design of planned studies.

## Main text

### Construction of pathway models of steroidogenesis using the mEPN notation

**Fig. 1:**
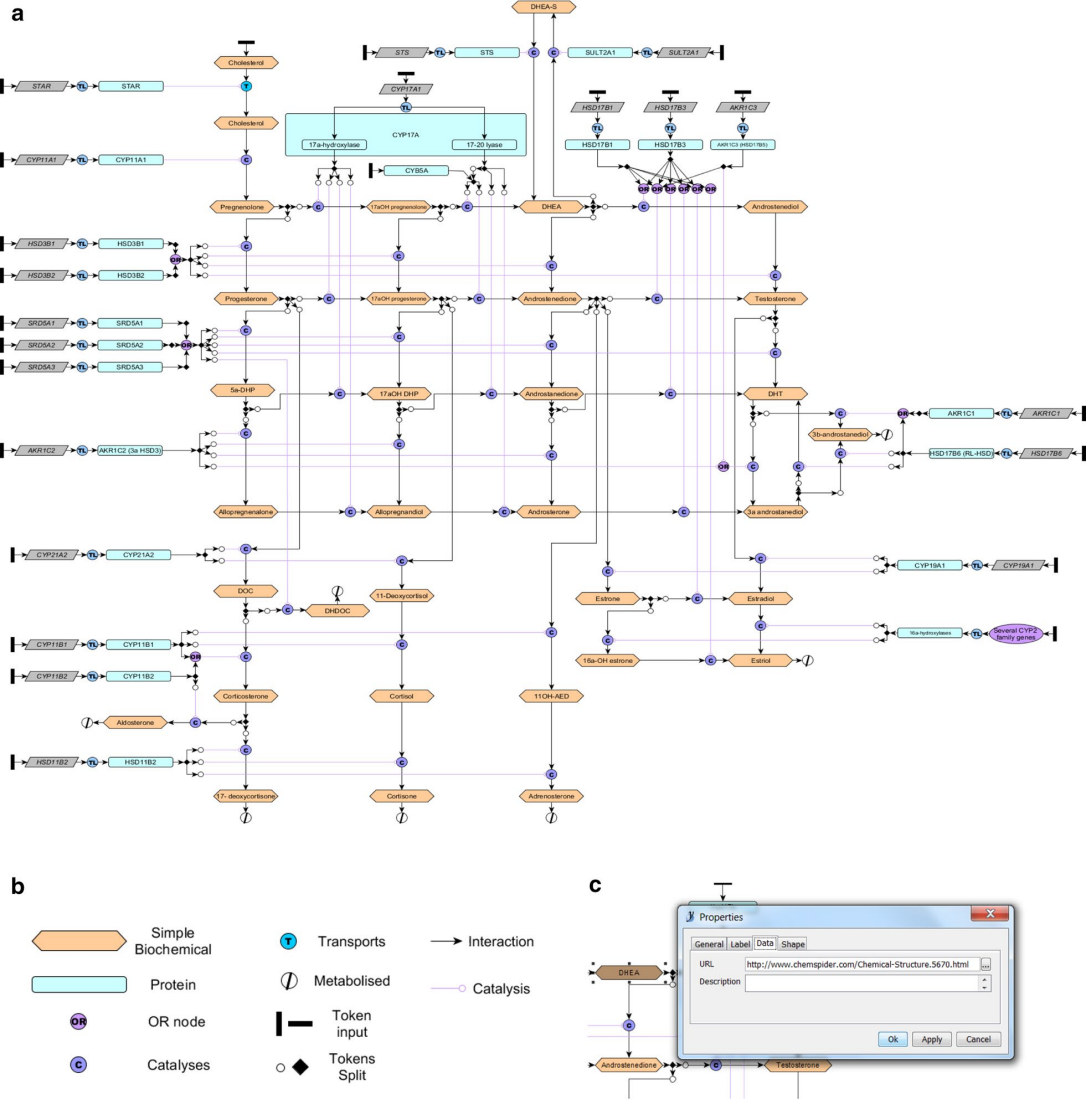
Construction of a framework model of human steroidogenesis. **a** An image of the editable framework model of human steroidogenesis. **b** A key to the symbols used in the diagram. **c** Addition of a Chemspider URL to a steroid node by editing the properties of the node in yED

A model of human steroidogenesis is presented in Fig. 1, representing a framework of the reactions that produce biologically active steroids under normal conditions. Diagrams were compiled using the modified Edinburgh pathway notation (mEPN) using the network editing software yED (http://www.yworks.com). The editable version of this diagram is available as a ‘.graphml’ file that can be opened in yED (Additional file 1). Steroid and enzyme interaction information was obtained from Miller et al. [1] and associated references. Active pathways in different tissues (namely the adrenal reticularis, glomerulosa and fasciculata, the testis, ovary, prostate and placenta, Additional file 2) are highlighted with red boxes to easily visualise the steroidogenic reactions that take place in each tissue.

mEPN is based on the principles of ‘process diagrams’ and is designed to be unambiguous yet concise [3]. A detailed protocol of how to edit mEPN diagrams has been recently published [4]. Both the biological entities (such as proteins or steroids) and the way they interact with each other (such as phosphorylation or dimerisation) are represented as components in the pathway. Small biochemicals such as steroids are represented by hexagons, all proteins (enzymes) by rounded rectangles and all genes by parallelograms. Black rectangles allow for parameterisation of models whereby initial token input on nodes feeding into the pathway can be defined (Fig. 1b). It can be expanded to produce large, clear and informative pathway models [5, 6].

We have chosen to label steroids and their intermediates based on names in common use in the biological community but have also linked nodes to the Chemspider database (http://www.chemspider.com). This provides a reference to the exact biochemical structure a molecule represents and gives alternate names. The link can be opened by pressing the F8 key when the node is highlighted in yED (Fig. 1c). There are number of naming conventions for biochemical molecules. The comprehensive diagram of the major steroidogenic pathways (Fig. 1a) contains nodes to represent both the gene and the protein produced for each enzyme isoform, and therefore each gene node is linked to Ensembl (http://www.ensembl.org) [7].

### Construction and parameterisation for dynamic flow of a cell-specific model of rat Leydig cell steroidogenesis using previously-published experimental data

**Fig. 2:**
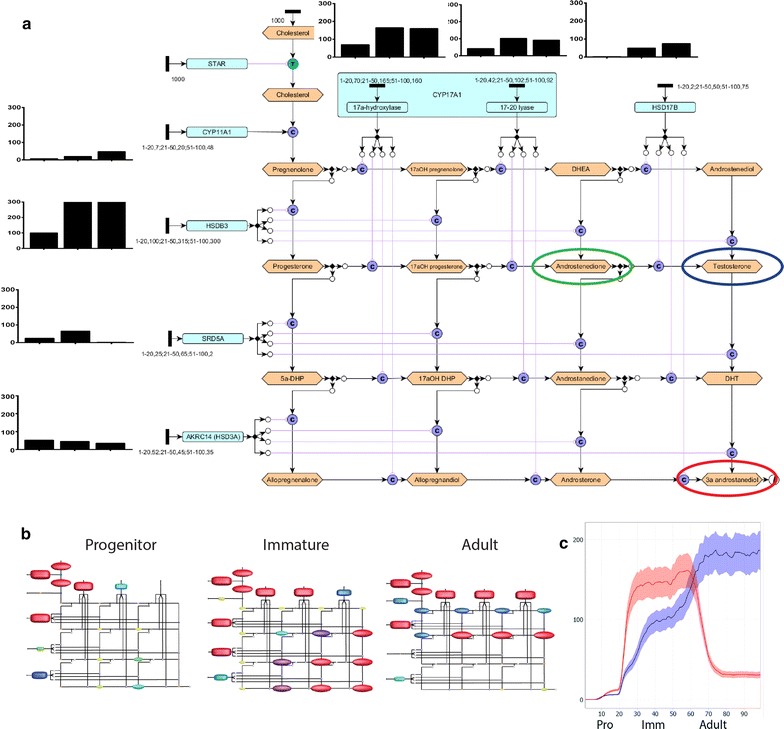
Construction, paramaterisation and hypothesis testing using a cell-specific model of rat Leydig cell steroidogenesis. **a** An image of the editable framework of rat Leydig cell steroidogenesis, with parameterisation details. The black bar graphs next to each enzyme input show the number of tokens added to each enzyme node at the three stages of progenitor, immature and adult Leydig cell. The tokens are introduced to the node by editing the properties of the arrow leading from the token input to the node, and are visible as a numerical string next to the node. Androstenedione is indicated by a green ring, testosterone in blue and 3α-diol in red. **b** Still frames from the dynamic signalling Petri net outputs of the Leydig cell model at the three stages of progenitor, immature and adult. Each of the coloured shapes on the diagram represents a steroid node in the same position as the yED diagram above. Larger red shapes indicate more tokens are flowing through that node, smaller blue shapes indicate fewer and no shape indicates no tokens. **c** A graph visualising the flow of tokens through the nodes representing testosterone (blue) and 3α-diol (red) over the course of Leydig cell maturation. The coloured areas around the line indicate the variance of the output

Whilst the formalised framework diagram has utility as a resource in its own right, the ability to parameterise the pathway and run and test simulations immeasurably adds to its overall value. We constructed and parametrised a specific model of rat Leydig cell steroidogenesis during postnatal development and adulthood (Fig. 2) focussing on the specific reactions that Leydig cells use to produce their main steroid product: androgens. The simplified Leydig cell version (Fig. 2a) uses a single protein node to represent all isoforms of a particular enzyme and so an Ensembl link is not included. The editable version of the simplified diagram is also available as a ‘.graphml’ file that can be opened in yED, (Additional file 3).

Parameterisation of pathway diagrams constructed in yED to run as a signalling Petri nets (SPNs) in the software Graphia Professional is a logical process and no formal training in mathematical modelling is necessary for the user. A detailed description of how to parameterise yED diagrams so that they can be run as SPNs in Graphia Professional (Kajeka, Edinburgh, UK, formerly BioLayout *Express*^3D^) [2] can be found in Livigni et al. [4]. By representing a complex network as a Petri net, the SPN method models signal flow as the pattern of token accumulation at protein nodes over time.

Parameterisation of the cell-specific diagram was achieved using previously published enzyme activity data measured at three different stages of rat Leydig cell maturation [8]. In yED, tokens representing the enzyme activity in pmol/minute/million cells were added to the arrows connecting the black input nodes of each of the steroidogenic enzyme nodes using the notation a–b,c;d–e,f where ‘a–b’ are the first and last time blocks that you would like the number of tokens ‘c’ to be added to the model and ‘d–e’ are the first and last time blocks that you would like the number of tokens ‘f’ to be added to the model (and so on for the number of variable inputs required), as shown in Fig. 2a. The first 20 time blocks represent the ‘progenitor’ Leydig cell stage present at around postnatal day (pnd) 21 in the rat. Time blocks 21–50 represent the ‘immature’ Leydig cell stage present at around pnd 35 and time blocks 51–100 represent the mature adult Leydig cell that constitute all of the Leydig cells in the testis from pnd 90 onwards. The variation in enzyme activity at the three stages is illustrated by the black bar graphs next to the enzyme input nodes in Fig. 2a. The editable version of the parameterised diagram is available as a ‘.graphml’ file that can be opened in yED (Additional file 4).

The parameterised diagram was run as a SPN in Graphia Professional over 100 time blocks, 500 runs and with standard normal stochastic distribution and consumptive transitions [2]. The token flow was visualised as an animation seen in Additional file 5. Figure 2b shows a screenshot of the animated output of the token flow at each of the three stages (screenshots taken at time block 20 representing progenitor, 50 representing immature and 100 representing adult Leydig cell stages). This provides an overview of all of the nodes in the diagram and helps visualise the pathways of token flow. Two nodes were selected for specific visualisation in Fig. 2c: testosterone and 3α-androstanediol (‘3α-diol’). If these outputs are taken as a prediction of the relative production rate of these two steroids at immature and adult Leydig cell stages, we would predict that 3α-diol is more abundant than testosterone in immature Leydig cells and that testosterone is more abundant than 3α-diol in adult Leydig cells. This prediction of the model is consistent with previous experimental measurement [8], showing that our model appropriately recapitulates the in vivo situation and thus has utility for hypothesis testing.

### Using the model to predict steroid production in Hsd17b3 deficiency

**Fig. 3:**
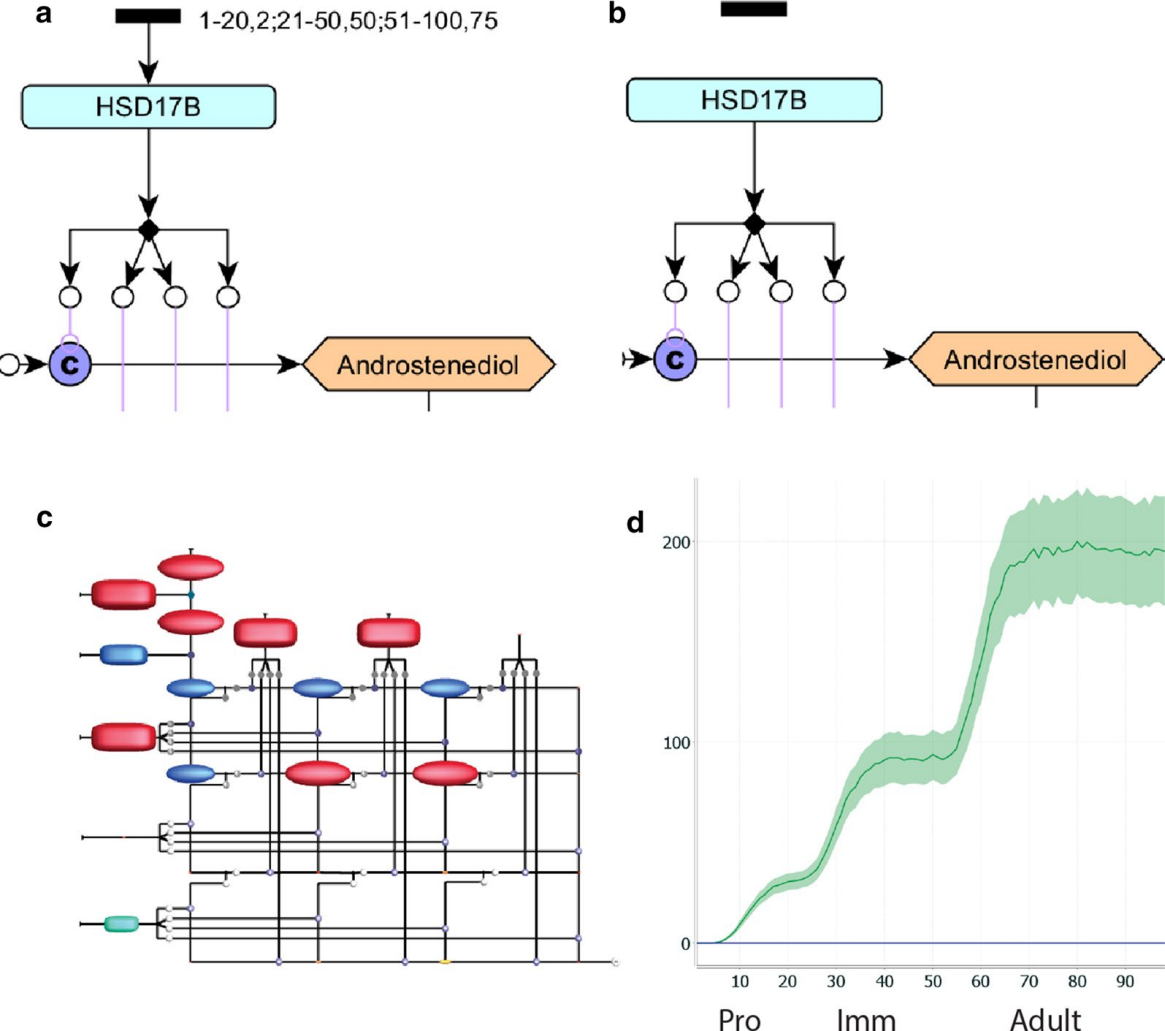
Using the model to predict the outcome of a HSD17B knockout in Leydig cells. **a** The labelling of the HSD17B protein activity node in a wild-type model. **b** The removal of the arrow between the token input and the protein node sets token input to zero and models a knockout of the protein. **c** A still frame from the dynamic signalling Petri net outputs of the HSD17B knockout Leydig cell model at the adult stage. Each of the coloured shapes on the diagram represents a steroid node in the same position as the yED diagram above. Larger red shapes indicate more tokens are flowing through that node, smaller blue shapes indicate fewer and no shape indicates no tokens. **d** A graph visualising the flow of tokens through the nodes representing testosterone (blue) and androstenediol (green) over the course of Leydig cell maturation. The coloured areas around the line indicate the variance of the output

Enzymes of the 17-beta hydroxysteroid dehydrogenase class catalyse the conversion between 17-keto and 17-hydroxy-steroids. Different isoforms of the enzyme are expressed in different steroidogenic tissues. 17-beta hydroxysteroid dehydrogenase type 3 is the isoform expressed by Leydig cells in humans (*HSD17B3*), mice and rats (*Hsd17b3*) [9] and preferentially catalyses the conversion of androstenedione to testosterone and androstanedione to DHT. Male humans with mutations in *HSD17B3* present with varying degrees of physiological undervirilisation and plasma androstenedione levels at the time of puberty are usually ten times normal levels [10]. To demonstrate the predictive power of our in silico model, we mimicked a loss of function mutation in *Hsd17b3* by removing the token input from the HSD17B node of the rat Leydig cell model (Fig. 3a, b), and re-ran the simulation. This time tokens accumulated at the androstenedione node, with no testosterone produced, consistent with the circulating androgen profile observed in patients with a loss of function of HSD17B3 (Fig. 3c). When visualised as a graph (Fig. 3d), androstenedione production is shown to increase as Leydig cells mature during postnatal life, whereas no testosterone is produced. This simple demonstration shows the utility of the model for predicting outcomes of genetic or pharmacological manipulations before beginning any laboratory or in vivo work, and has the potential to be scaled with multiple knockouts modelled simultaneously.

## Limitations

The system we describe here presents many significant advantages over previous modelling systems. The software is free and readily available, and is supported by two recent publications explaining the underlying methodology [2], and specific protocols that describe the editing of existing models and construction of new models. In its graphical form within yED, specific nodes within a diagram can be hyperlinked to publications describing the supporting evidence and to references to correct gene and chemical nomenclature. As such the diagram can represent a visual bibliography of known interactions and supporting data, and we have found this to be a much-appreciated resource by anyone grappling to conceptualise the complexities of the endocrine system.

The true power of the framework diagram is revealed when combined with stochastic modelling within Graphia Professional. Traditionally, systems dynamics are described using continuous deterministic mathematical models, which assume that the system has no unpredictability and that the precise behaviour of its components is entirely pre-determined. However, biological systems are intrinsically stochastic and there is evidence that stochasticity is advantageous [11]. In this case, Petri nets, which are a mathematical modelling language for the description of distributed systems, allow for the study of dynamics without the need to have detailed information on the kinetics. It also means that the system is significantly more ‘biologist friendly’ than mathematical modelling through ordinary differential equations.

The system can be used where information is missing, as it is possible to substitute a single arrow (edge) to represent an uncharacterised event between two established known molecules, which permits modelling to continue without possession of all information. Thus, some areas of the model may be incredibly detailed, whilst others are described in less detail. This may identify areas and components that are missing, but must be necessary, thereby focussing hypothesis generation and laboratory experiments in these key locations to refine understanding.

In conclusion, the development of this framework model of steroidogenesis using free software to edit and construct new models will support in silico hypothesis generation and testing across many endocrine endpoints. Use of this system has significant potential to reduce costs, time and animal numbers, whilst informing the design of planned studies.

## Additional files

**Additional file 1.** Editable Graphml file of a framework model of human steroidogenesis.

**Additional file 2.** Active steroidogenic pathways in selected steroidogenic tissues highlighted on the framework model.

**Additional file 3.** Editable Graphml file of a framework model of rat Leydig cell steroidogenesis.

**Additional file 4.** Editable Graphml file of a parameterised model of rat Leydig cell steroidogenesis.

**Additional file 5.** Animation demonstrating token flow through a paramterised model of rat Leydig cell steroidogenesis in Graphia Professional.

## Abbreviations

11-DOC: 11-deoxycorticosterone
16OH-estrone: 16α-hydroxyestrone
17α-OH DHP: 17α-hydroxy dihydroprogesterone
3α-diol: 3α-androstanediol
5α-DHP: 5α-dihydroprogesterone
Cyp11a1: cytochrome p450 side-chain cleavage
DHDOC: dihydrodeoxycorticosterone
DHEA: dehydroepiandrosterone
DHT: dihydrotestosterone
DOC: deoxycorticosterone
Hsd17b(3): 17β-hydroxysteroid dehydrogenase (type 3)
mEPN: modified Edinburgh pathway notation
pnd: post-natal day
SPN: signalling Petri net
