# Supplementary material for: Modelling steroidogenesis: a framework model to support hypothesis generation and testing across endocrine studies
Source: BMC Res Notes. 2018 Apr 24;11:252. doi: 10.1186/s13104-018-3365-y (PMC5937803; doi:10.1186/s13104-018-3365-y)

Additional file 3

Adrenal glomerulosa


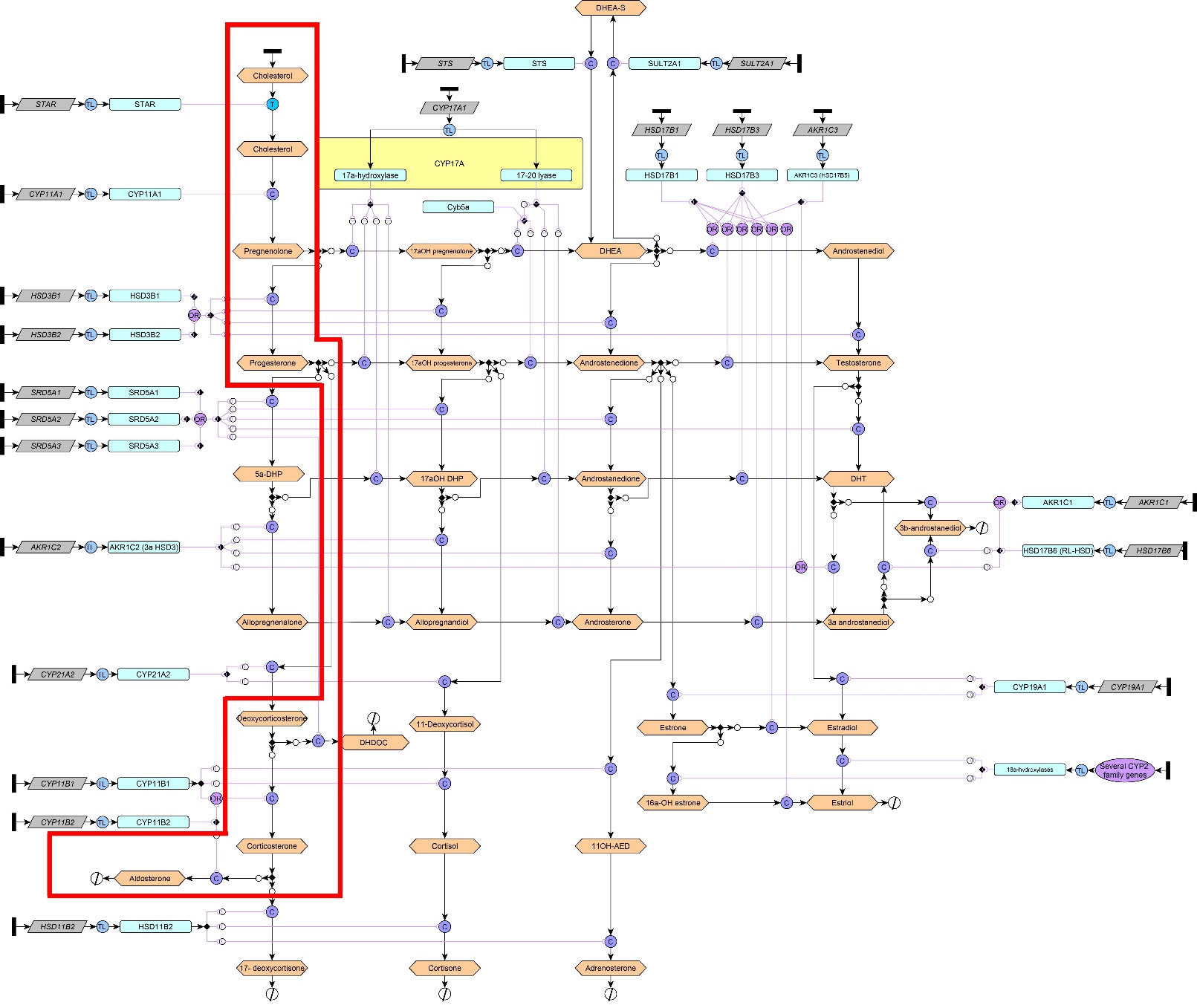


Prostate


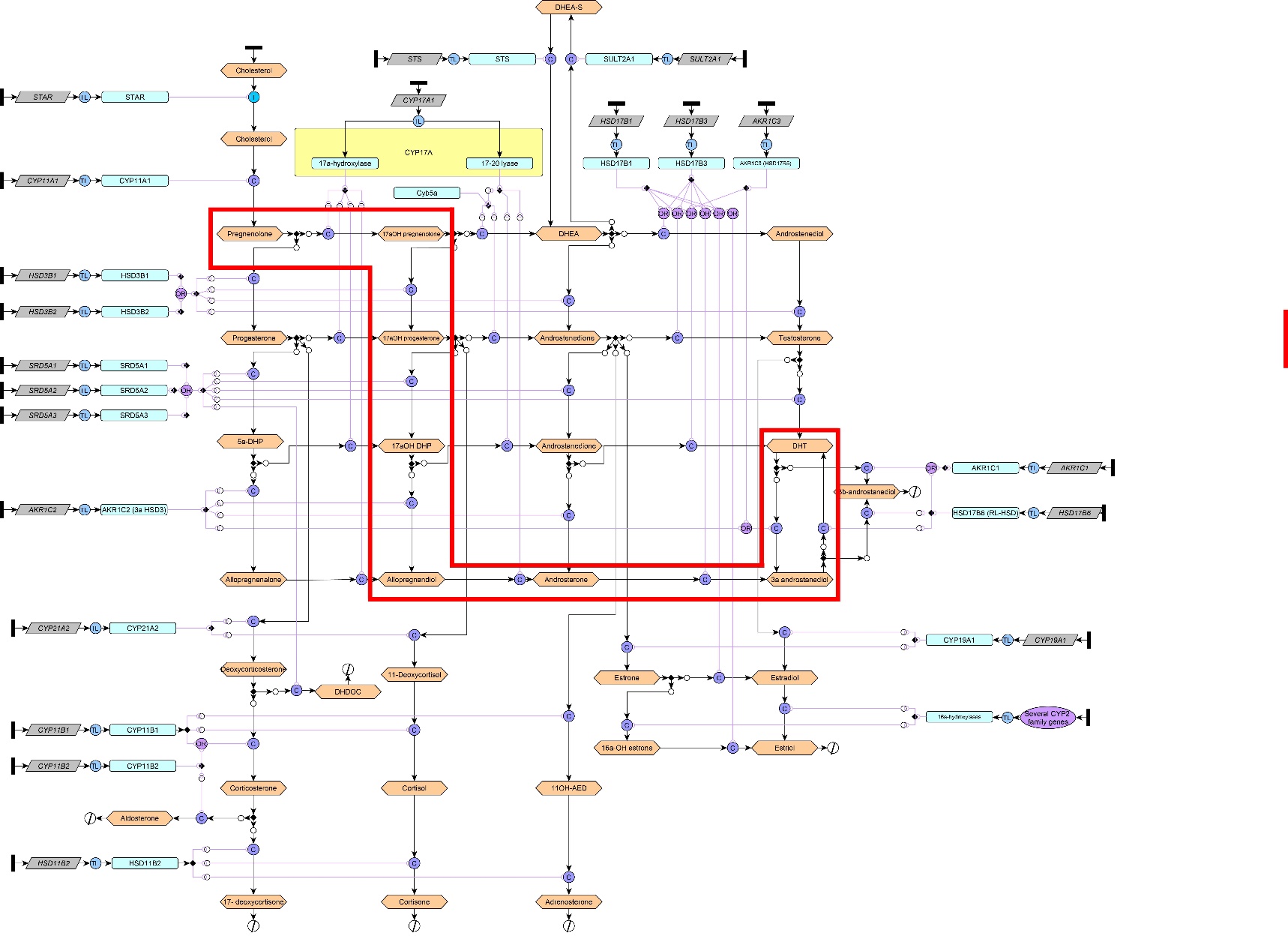


Adrenal fasciculata


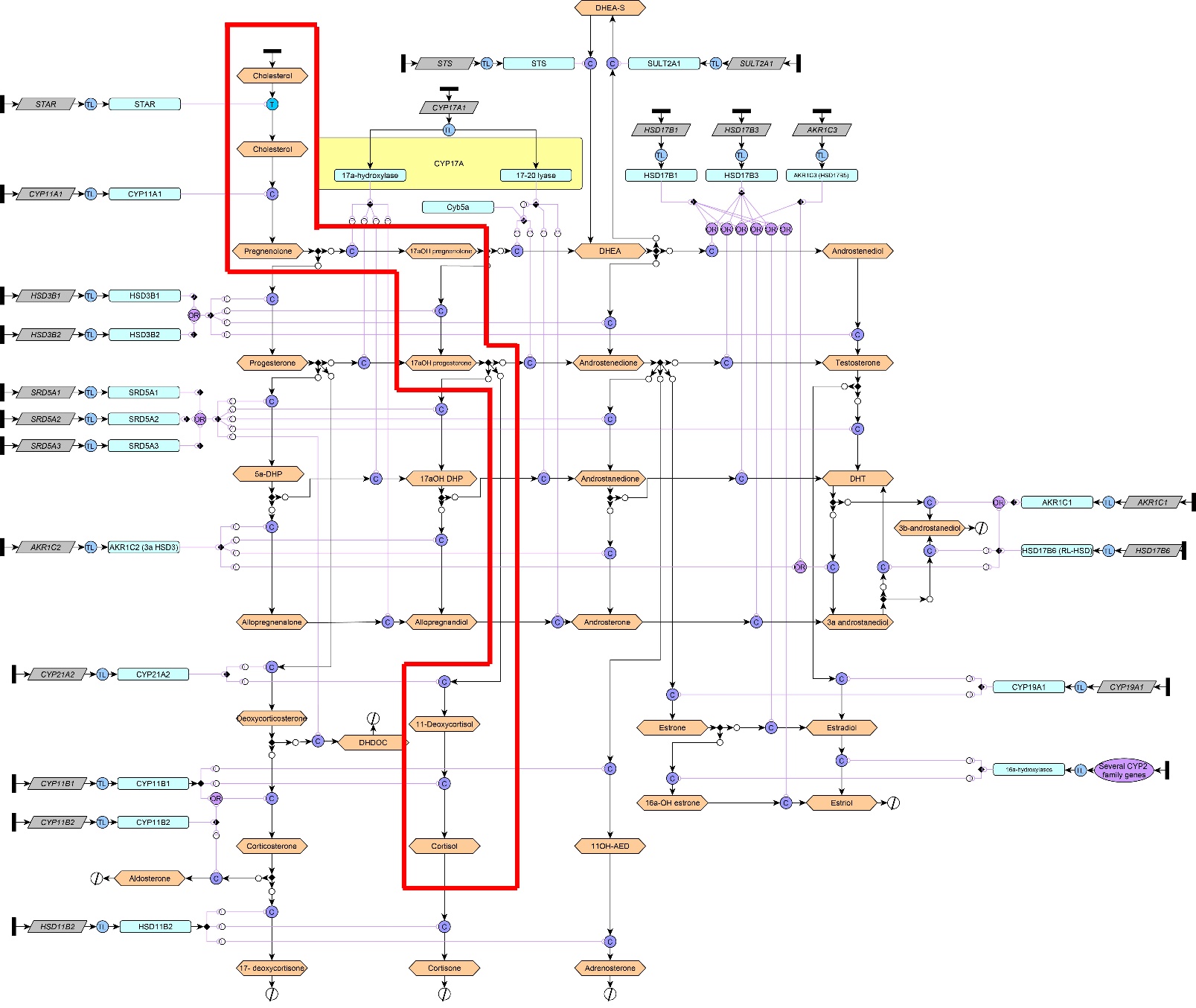


Ovary


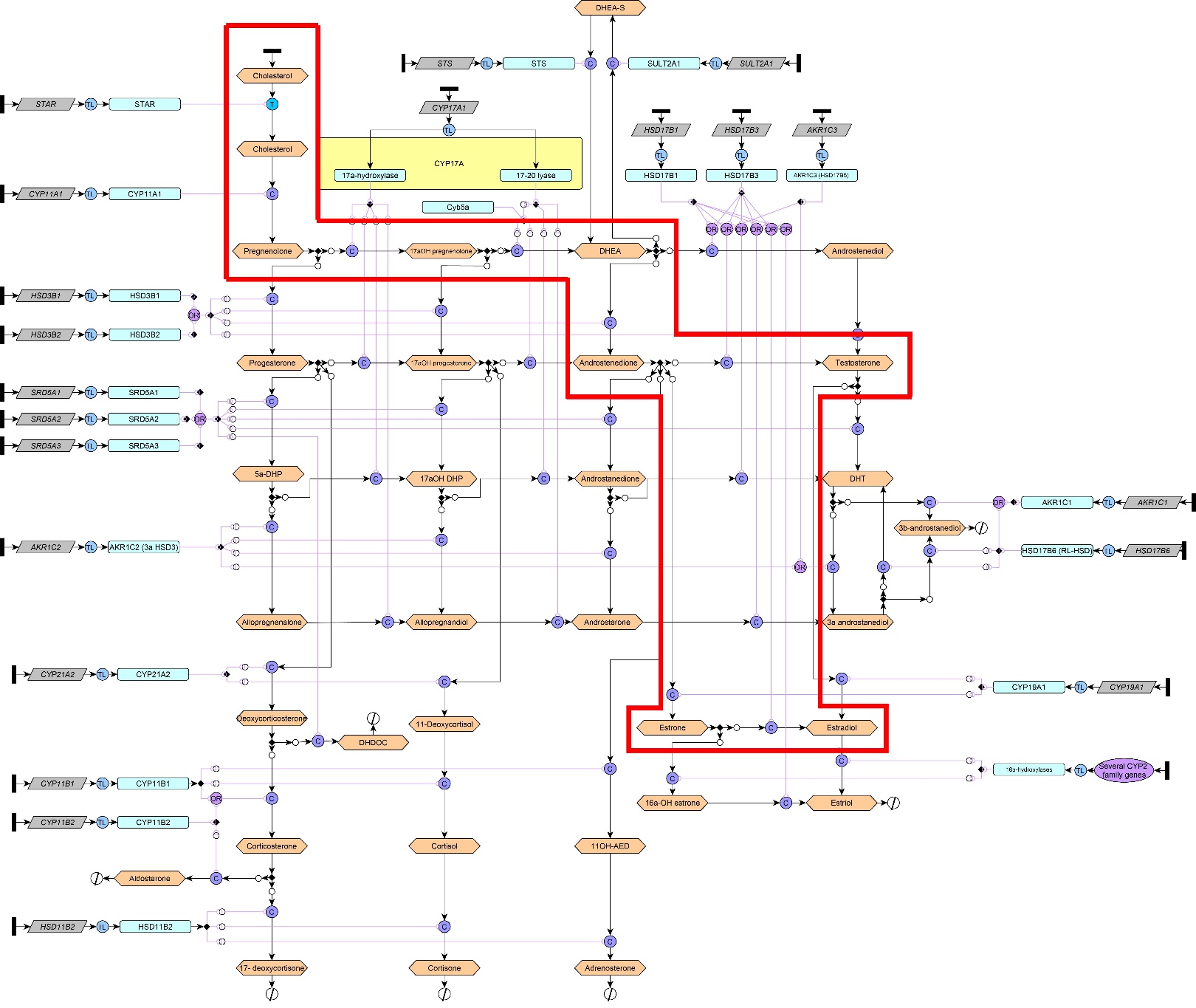


Testis


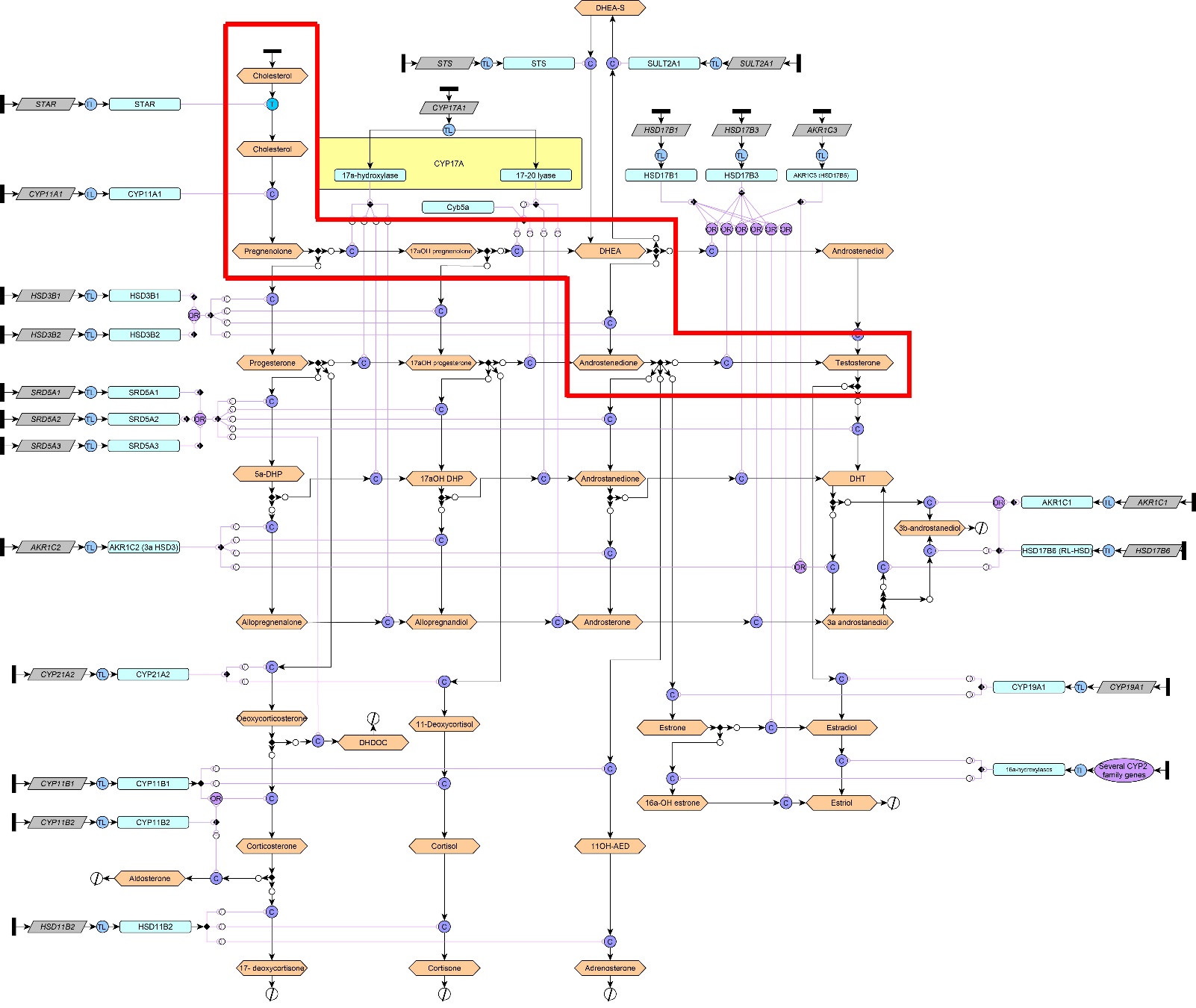


Adrenal reticularis


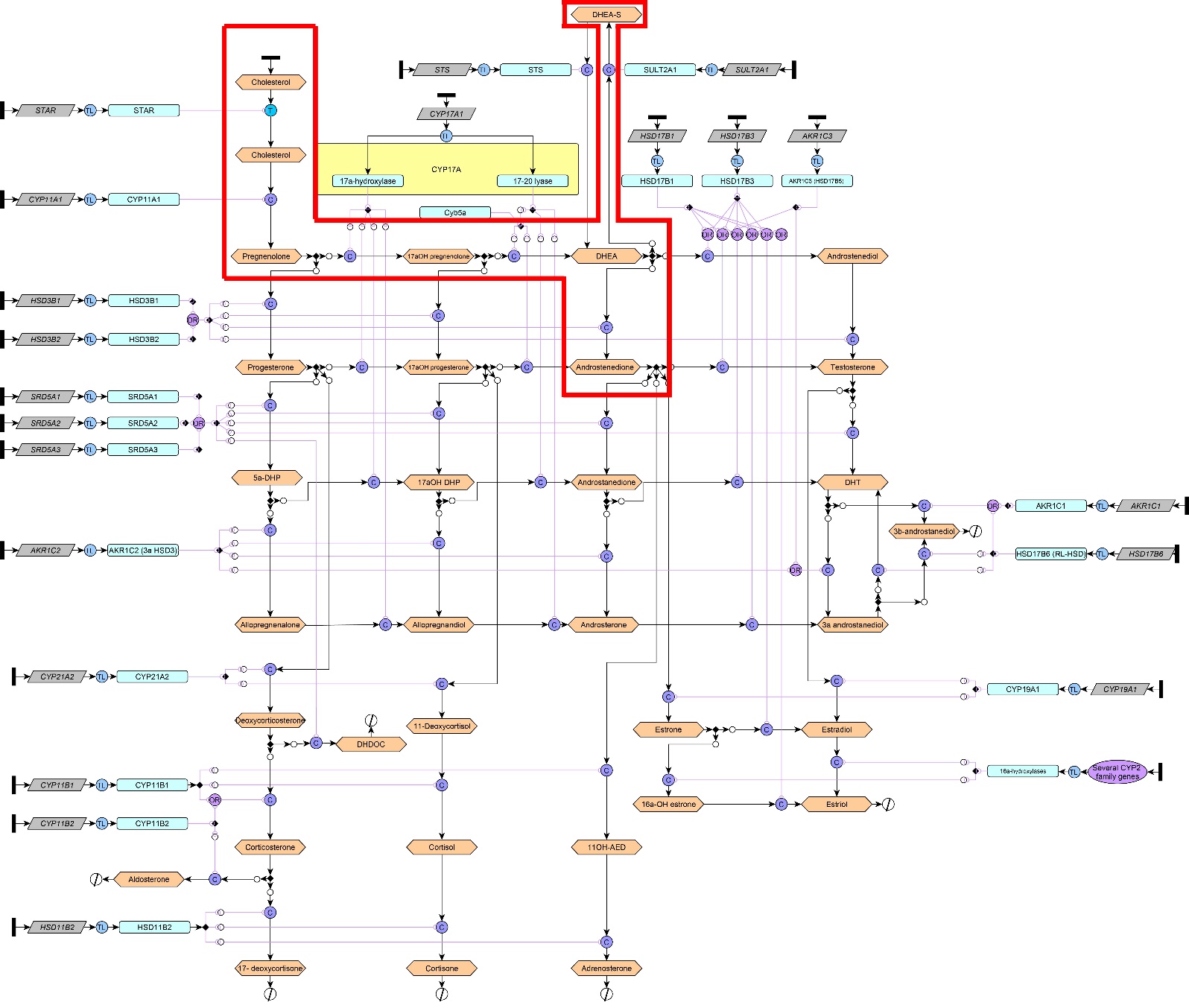


Placenta


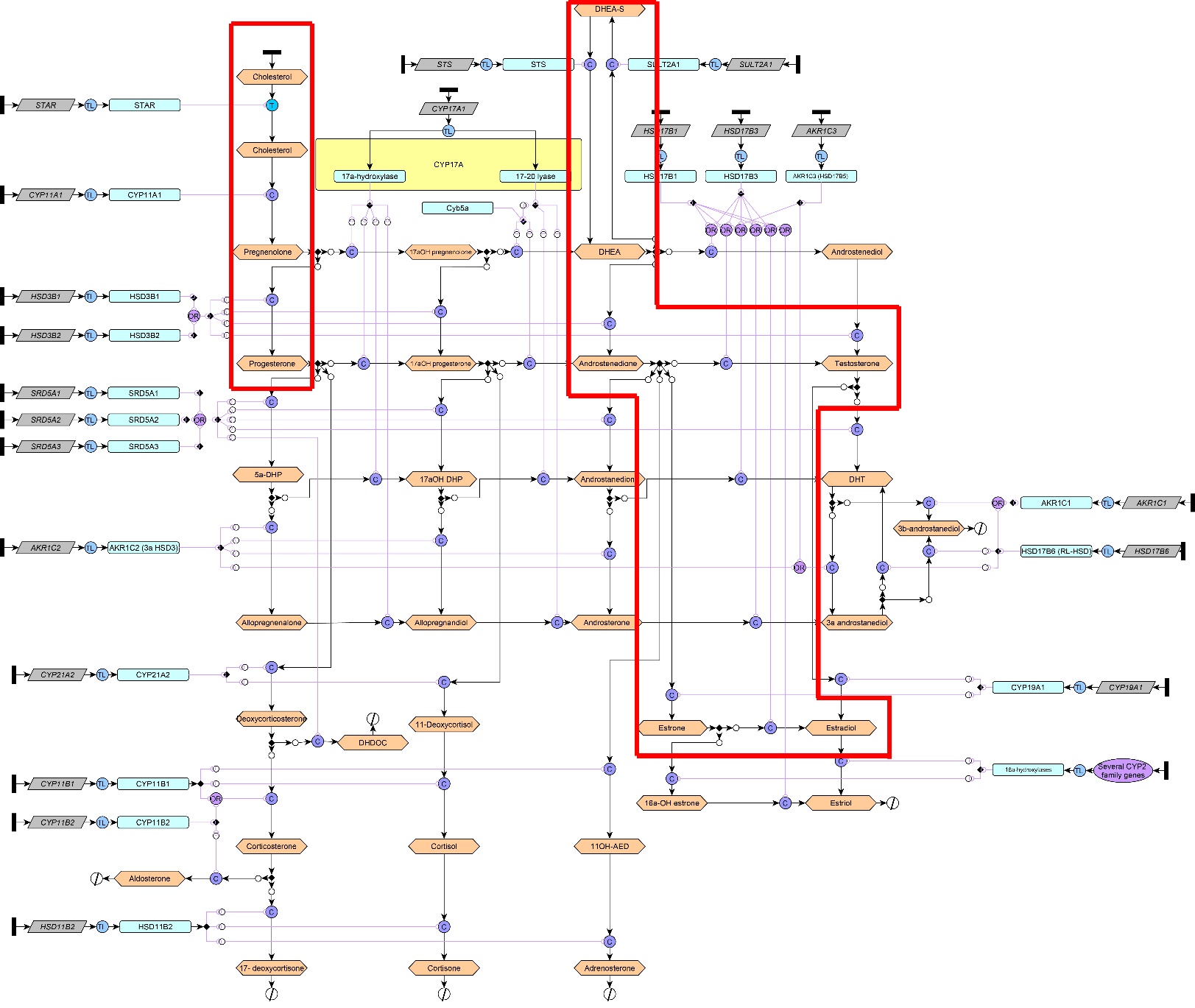

Supplement: Supplementary file 2 — Additional file 2. Active steroidogenic pathways in selected steroidogenic tissues highlighted on the framework model. [file 13104_2018_3365_MOESM2_ESM.docx]
